# Modulation of CYP2E1 metabolic activity in a cohort of confirmed caffeine ingesting pregnant women with preterm offspring

**DOI:** 10.1186/s40348-020-00096-3

**Published:** 2020-06-01

**Authors:** M. R. Alcorta-García, C. N. López-Villaseñor, G. Sánchez-Ferrer, H. Flores-Mendoza, F. Castorena-Torres, M. A. Aguilar-Torres, C. M. Sepúlveda-Treviño, J. A. Hernández-Hernández, R. C. López-Sánchez, V. J. Lara-Díaz

**Affiliations:** 1 Tecnologico de Monterrey, Escuela de Medicina y Ciencias de la Salud, Avenida Ignacio Morones Prieto 3000 poniente, Colonia Doctores, CP 64710 Monterrey, Nuevo León México; 2 Hospital Regional Materno Infantil, Secretaria de Salud, Gobierno del Estado de Nuevo León, Avenida San Rafael 450, Colonia San Rafael, CP 67140 Ciudad Guadalupe, Nuevo León México

**Keywords:** Infant, Premature, Premature birth, Caffeine, Cytochrome P-450 Enzyme System, Cytochrome P-450 CYP1A2 inducers, Cytochrome P-450 CYP1A2 inhibitors, Cytochrome P-450 CYP2E1 inducers, Cytochrome P-450 CYP2E1 inhibitors

## Abstract

**Background:**

To ascertain interactions of caffeine ingestion, food, medications, and environmental exposures during preterm human gestation, under informed consent, we studied a cohort of Mexican women with further preterm offspring born at ≤ 34 completed weeks. At birth, blood samples were taken from mothers and umbilical cords to determine caffeine and metabolites concentrations and CYP1A2 (rs762551) and CYP2E1 (rs2031920, rs3813867) polymorphisms involved in caffeine metabolism.

**Results:**

In 90 pregnant women who gave birth to 98 preterm neonates, self-informed caffeine ingestion rate was 97%, laboratory confirmed rate was 93 %. Theobromine was the predominant metabolite found. Consumption of acetaminophen correlated significantly with changes in caffeine metabolism (acetaminophen *R*^2^ = 0.637, *p* = 0.01) due to activation of CYP2E1 alternate pathways. The main caffeine source was cola soft drinks.

**Conclusion:**

Environmental exposures, especially acetaminophen ingestion during human preterm pregnancy, can modulate CYP2E1 metabolic activity.

## Background

Caffeine, one of the naturally occurring methylxanthines, is the most widely consumed behaviorally active substance in the world. Almost all caffeine comes from dietary sources (beverages and food), most of it from coffee and tea, but some other sources, like soft drinks, are showing increased prevalence. Of all the psychoactive drugs, caffeine is indeed the most widely used [1].

The majority of expecting mothers in the Western world drink beverages containing methylxanthines during pregnancy, and they continue their consumption during lactation. Thus, many fetuses and infants are exposed to these substances. In addition, a large number of premature infants need pharmacological treatment for apnea of prematurity with methylxanthines [2]. Existing data suggests that caffeine consumption at doses below 300 mg/day and perhaps up to 400 mg/day during pregnancy, beyond the first trimester of pregnancy, does not increase the risk of any reproductive or perinatal adversity [3].

Maternal caffeine consumption during pregnancy has been studied for many years, but convincing evidence for an association with poor perinatal outcomes remains elusive [4].

Caffeine metabolism is substantially slowed during pregnancy, mainly due to a reduction in CYP1A2 (CYP1A2, cytochrome P450 family 1 subfamily A member 2) and N-acetyltransferase-2 (NAT2) activities. NAT2 metabolic ratio is lower in early pregnancy than after delivery; CYP1A2 activity decreases not only in late pregnancy but also in early and middle pregnancy, while the ratio of 8-hydroxilation increases in both middle and late pregnancy [5–8].

Thus, under normal pregnancy conditions, caffeine and its metabolites readily cross the placental barrier, exposing the fetus for a prolonged time, since neither the fetus nor the placenta have caffeine metabolic capabilities [9].

For technical, legal, and ethical reasons, it is difficult to perform studies on drug disposition in pregnant women. However, for rational drug use, we need more information on drug disposition during pregnancy [5]. Non-interventional studies, as ours, offer a unique opportunity to attain this goal.

Most studies have used maternal self-reported caffeine consumption to estimate fetal exposure to caffeine during pregnancy [10] and the vast majority have focused on term pregnancies, besides, there are not specific studies on the cytochrome metabolic activity, genotyping and the effect of exposure to potential inducers or modifiers of these enzymatic processes in the preterm pregnancy.

Additionally, some CYP1A2 and CYP2E1 (CYP2E1, cytochrome P450 family 2 subfamily E member 1) polymorphisms exhibit different caffeine metabolism, as rs762551 (CYP1A2*1F), rs3813867 (CYP2E1*5A), and rs2031920 (CYP2E1*5B). Environmental conditions such as smoking, exposure to aromatic hydrocarbons (tobacco smoke, pollution, and occupational exposure to carbon smoke), along with the ingestion of food with nitrosamines (charbroiled meat) are modifiers of CYP1A2 activity. Moreover, the inhalation of volatile aromatic hydrocarbons as well as the consumption of alcohol and intake of prescribed or over-the-counter medications allowed in pregnancy, further modify cytochrome activity and can even be inducers or substrates of CYP2E1 [11–13].

In this study, we explored patterns of caffeine consumption and transplacental transfer of caffeine and its metabolites, in a cohort of expecting mothers who gave birth to preterm neonates below 34 weeks of gestation, and searched for correlations of CYP1A2 and CYP2E1 isoforms [12–14], the maternal caffeine metabolic activity and certain environmental, health and life-style conditions concurrent to pregnancy. This maternal population was selected because of the high probability that their preterm offspring would be in need of postnatal caffeine administration, in order to treat apnea of prematurity, particularly frequent in this neonatal condition [15]. Our main interest was to identify any changes in the maternal caffeine metabolic activity that could further modify their fetuses caffeine metabolic activity once born.

## Patients and methods

### *Patients*

We conducted this prospective cohort study from September 2013 to August 2014, in two medical centers located in Northeastern Mexico: The Hospital Regional Materno-Infantil (Center 1) and the Hospital Metropolitano Dr. Bernardo Sepúlveda (Center 2). These two public hospitals have level II neonatal intensive care units and belong to the Servicios de Salud Network in Nuevo León, México.

The institutional human research review boards at Escuela de Medicina y Ciencias de la Salud, Tecnologico de Monterrey and at both study hospitals revised and approved the study protocol, with the identifier CAF-CYP1A2. All laboratory procedures were performed in accordance with relevant guidelines and regulations.

Dedicated house officers screened eligible mothers with an imminent delivery before 34 completed weeks, and invited them to participate; a written informed consent was obtained from each participant before giving birth. The assessment of gestational age was done preferably through ultrasonography in the first weeks of fetal life or, if such data were unavailable, calculated upon the date of last menstrual period, and confirmed through clinical examination of the neonate. Birth weight was measured using an electronic infant weighing scale (Seca 354®), and the neonates were classified according to Fenton´s growth charts [16].

Infants were included if a member of the research team was present at delivery, no gender or birth weight cutoff was considered for enrollment. We excluded those infants whose mothers refused consent, those who had major birth defects at delivery, those who had a delayed birth and those born to mothers under xanthines, proton pump inhibitors, antifungals, or antiretroviral therapy.

Complete medical histories were conducted, and a detailed questionnaire was administered by one of the researchers. The questionnaire asked women to report beverage intake over the past 3 months. Detailed information was collected specifically on the ingestion of regular coffee, decaffeinated coffee, espresso coffee or coffee mixed with milk, regular tea, herbal or green tea, energy drinks containing caffeine, chocolate, and bottles of cola soft-drinks (sweetened and diet separately), and other soft-drinks without caffeine (sweetened and diet separately) consumed each week. It also asked for information on nutritional habits, exposure to alcohol, tobacco, and medications with potential to interact with CYP1A2 and/or CYP2E1, and environmental contaminants, during on-course pregnancy, with a special emphasis on the trimester immediately prior to delivery. The questionnaire’s validity had been previously assessed through discussion groups with attending neonatologists and neonatal nurses, as well as with pregnant women focus groups [unpublished data]. In order to gather other relevant data not obtained during interview, we consulted electronic medical records.

### Blood sampling and labeling

Maternal and cord blood for caffeine and metabolite quantification were drawn pre and post-birth respectively. A sample of 4.5 mL of venous maternal blood was obtained by peripheral puncture, into a 13 × 100 mm BD Vacutainer® PPT-K2™ gel tube; cord blood was obtained by puncture of cord vessels and transferred to a 13 × 100 mm BD Vacutainer® PPT-K2™ gel tube for the same purpose. Samples were labeled for identification and transferred immediately to the main research facility, where they were centrifuged for 5 minutes at 8,000 rpm for plasma separation. Plasma was then stored at – 80 °C until analysis.

### Caffeine and metabolites quantification

Maternal and cord blood separated frozen plasma was thawed for caffeine and metabolites quantification. A rapid and selective high-performance liquid chromatographic (HPLC) method was used for the separation and determination of caffeine, paraxanthine, theobromine, theophylline, and our internal standard 7-beta-hydroxiehtyl-theophylline [17]. All analytical grade solvents and reagents were purchased from VWR®. Solid phase extraction including conditioning, pretreatment, sample introduction, washes and elution were done according to literature and supplier (Phenomenex®) specifications.

Trans placental transfer in vivo: The simplest index for the in vivo determination of trans placental transfer of a drug is a ratio of fetal to maternal blood concentrations, known as F/M ratio [18].

### CYP1A2 and CYP2E1 polymorphisms

DNA extraction: genomic DNA was extracted in accordance with FTA*™* card (Taqman*™,* GE Healthcare Life Sciences) method. Both maternal and cord blood droplets were placed in the FTA*™* cards at the same time that venipuncture was performed for clinical tests withdrawal. FTA*™* cards were labeled and transferred to the research facility. Once there, samples were allowed to dry and then, stored in aluminum foil under humidity-controlled conditions until extraction. For genomic DNA extraction, FTA cards were hole-punched twice to obtain an adequate sample; afterwards, the punched fragments were placed in a tube, and 3 to 4 washes were performed using FTA*™* purification reagent (Taqman*™,* GE Healthcare Life Sciences). Following these washes, DNA was re-suspended with the addition of preheated Tris-(Hydroxymethyl)-Aminomethane (Tris) and sodium hydroxide (NaOH). Tubes were then labeled, identified, and stored at – 20 °C.

### Determination of single-nucleotide polymorphism

Genetics analysis included the determination of three variants with clinical relevancy: *CYP1A2* (*rs762551*), and the variants of *CYP2E1* (*rs3813867, rs2031920*) [19, 20].

Allelic discrimination was performed by real-time polymerase chain reaction with specific primers and Taqman® probes. Briefly, approximately 50 ng of genomic DNA was added to a master mix (Applied Biosystems, Foster City, CA, USA) containing 200 nmol of primers and 40 nmol of probes for each polymorphism. Samples were set in a 96-well plate and amplified as follows: pre-PCR read at 60 °C for 30 s, then 10 min 95 °C, 35 cycles 15 s at 95 °C and 60 °C 1 min. Post-read analysis was performed at 60 °C for 30 s. All assays were carried out in a 7500 Fast PCR System (Applied Biosystems) and data analysis were performed with 7500 Fast System SDS software (ver 1.4.0.25).

### Cytochrome metabolic activity

CYP1A2 metabolic activity was assessed through the nanomolar conversion ratio of caffeine to paraxanthine, CYP2E1 metabolic activity was assessed by the nanomolar conversion ratio of caffeine to theobromine and the nanomolar conversion ratio of caffeine to theophylline, as has been published elsewhere [5, 20].

### Statistical analysis

The data were tabulated in Excel for Microsoft Office©; and analyzed using the statistical package IBM® SPSS® Statistics v24 for Microsoft Windows 10, Enterprise Edition©. Continuous variables with a normal distribution are expressed as means and standard deviations, otherwise, they are expressed as medians and interquartile ranges (IQR); categorical variables are expressed as frequencies and proportions. Differences were explored with Median test, Mann-Whitney’s *U* test, Kolmogorov-Smirnov’s test or Fisher’s exact test, as required. We did a descriptive analysis, followed by a correlation analysis between maternal blood caffeine concentration, exposure to medications, environmental exposures, clinical events concurrent to pregnancy, and CYP1A2*1F, CYP2E1*5A, and CYP2E1*5B polymorphisms presence. Variables with significant correlation were further tested in a linear regression model.

## Results

**Fig. 1:**
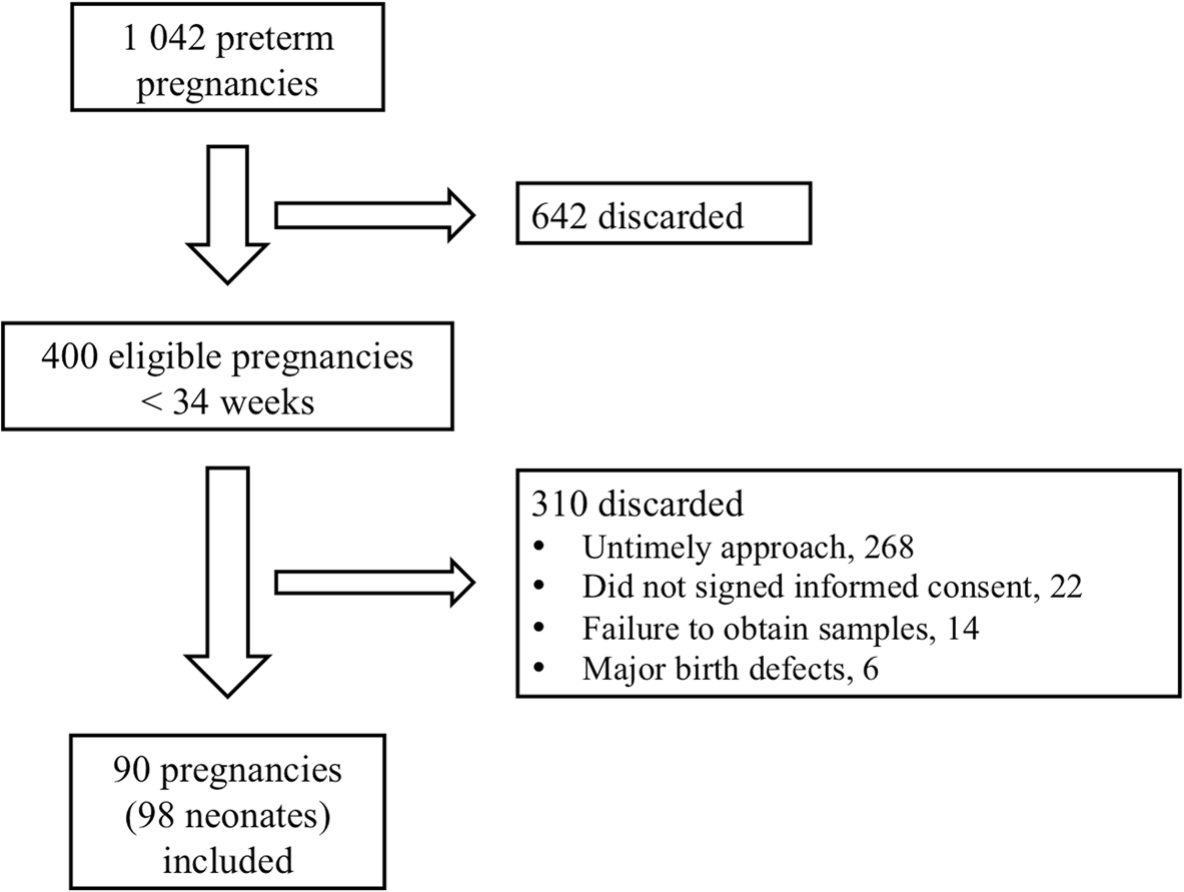
Study flow chart

Over the 12 months of the study period, 21,072 live births occurred at the study hospitals, 1042 were preterm and 400 were < 34 weeks (eligibility group). In Fig. 1, we illustrate the patient flow and eligibility process. We enrolled in the study 90 pregnancies, of which 8 (8.8%) were multiple pregnancies. These pregnancies gave birth to 98 preterm neonates, of which 55% were male, and 45% were female. These recruitment rates are consistent with hospital-based recruitment for a protocol needing a high commitment from participants [21]. In Table 1, we detail the study population characteristics.

**Table 1:** Clinical characteristics, lifestyle and habits of study population

Maternal characteristics, *n* = 90	Value
Maternal age, years ^a^	23.8 (6.6)
Gestational age, weeks ^a^	29.9 (2.6)
Gestational diabetes ^b^	5 (5.5)
Hypertension ^b^	10 (11)
Prenatal steroids ^b^	68 (76)
**Environmental exposure in pregnancy**	Value
Alcohol ^b^	1 (1)
Tobacco ^b^	None
Acetaminophen ^b^	6 (6.7)
Charcoal fumes ^b^	5 (5.5)
Charbroiled meat ^b^	46 (51)
**Neonatal characteristics, *n*= 98**	Value
Gender ^b^	
Male	54 (55)
Female	44 (45)
Mode of delivery^b^	
Vaginal	37 (38)
Abdominal	61 (62)
Birth weight (g) ^a^	1287 (407)
Birth length (cm) ^a^	38.1 (4.5)
Head circumference (cm) ^a^	27 (2.6)
Apgar 1 min^c^	7 (6 to 8)
Apgar 5 min^c^	9 (8 to 9)

^a^Mean (*SD* standard deviation)

^b^Frequency (proportion)

^c^Median (*IQR* interquartile range)

**Fig. 2:**
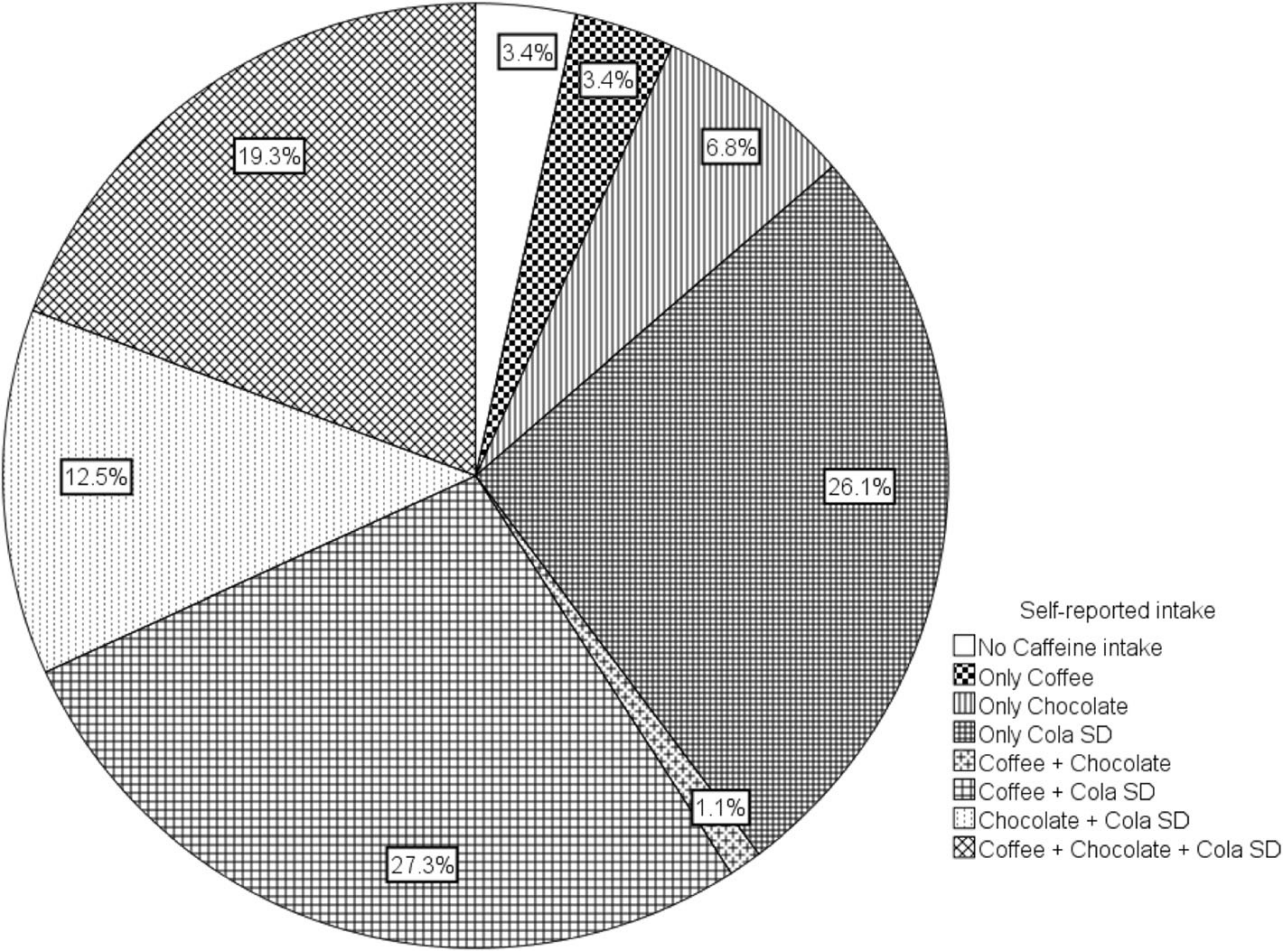
Sources of ingested caffeine in pregnant women with laboratory confirmed caffeine and metabolites in plasma

Caffeine consumption (from any known source) was self-reported in 87 of the 90 pregnancies evaluated (96.7%) and confirmed by detection of caffeine or any of its metabolites in blood in 84 of 90 mothers (93%). Women in this cohort had a tendency to over-report in regard to caffeine consumption, the kappa coefficient, 0.071, SE = 0.102, with a 95% confidence interval from − 0.132 to 0.274, identified a slight agreement between self-report and laboratory quantification. The sources of ingested caffeine are detailed in Fig. 2. Overall, the most common source of caffeine during pregnancy was coffee and cola soft drinks together, followed by cola soft drinks alone; these two sources accounted for more than half of caffeine during pregnancy. Cola soft drinks alone and in all possible combinations accounted for more than 85% of the caffeine sources in our population. Coffee alone accounted for only 5% of ingested caffeine, and coffee and combinations rose to 55% of caffeine sources. No instances of black tea, green tea, and mate herb or guarana consumption were reported in our cohort.

We measured the concentrations of caffeine, theobromine, theophylline and paraxanthine in maternal and cord (fetal) blood, in samples obtained immediately before (maternal) and after birth (cord blood). Caffeine and theobromine were the most abundant in maternal as well as in fetal blood; as expected due to the already known metabolic changes during pregnancy [5–8], average caffeine concentration in fetal blood was slightly higher than maternal concentration. Paraxanthine and theophylline concentrations resulted very similar in both compartments. Details on concentrations are offered in Table 2.

**Table 2:** Plasma concentration of caffeine and its main metabolites*

Source	Caffeine *n* = 84	Paraxanthine *n* = 81	Theobromine *n* = 84	Theophylline *n* = 84
Maternal plasma	1.18 (1.15)	0.12 (0.17)	0.46 (0.47)	0.08 (0.11)
Neonatal plasma	1.28 (1.37)	0.16 (0.20)	0.47 (0.60)	0.12 (0.12)

*μg/mL, mean (*SD* standard deviation). In all comparisons of caffeine and metabolite concentrations, no statistical difference was found (*t* test) between maternal and fetal compartment

The transplacental transfer ratio (F/M ratio) for caffeine was calculated as suggested [18, 22], 84 mother-infant pairs had suitable data. For the entire cohort, the mean caffeine F/M ratio was 1.17, SD 0.60, while the range went from 0.04 to 3.3. A low caffeine F/M ratio (up to a ratio of 1.0) was found in 31 pregnancies (37.3%), while a normal caffeine F/M ratio (above 1.0) was seen in 52 pregnancies (62.7%). A moderate positive correlation was observed between normal caffeine F/M ratios and positive genotyping of the homozygote-mutated allele of *CYP1A2*, Spearman´s Rho 0.251(79), *p* = 0.026; further exploration with *χ*^*2*^ test disregarded this association. A moderate negative correlation was observed for the occurrence of gestational diabetes mellitus and a low F/M ratio, Spearman’s Rho − 0.251(83), *p* = 0.022; further exploration with Fisher’s exact confirmed this association. No associations were observed among F/M ratios and the sources of ingested caffeine, gender of the newborn, occurrence of pregnancy-associated hypertension, exposure to antenatal steroids, via of delivery, exposure to acetaminophen, charbroiled meat, or charcoal fumes during pregnancy. Furthermore, caffeine F/M ratios did not exhibit correlation with time elapsed since the last ingestion of caffeine before birth.

### Genotype analysis

We selected polymorphisms that are relevant in the clinical practice [18]. The genotype distribution and allele frequency of CYP1A2*1F (*rs762551*) and *CYP2E1* polymorphisms (CYP2E1*5A rs3813867 and CYP2E1*5B rs20131920) are shown in Table 3.

**Table 3:** Frequency (percentage in parentheses) of allelic polymorphisms of CYP1A2 and CYP2E1 in pregnant women who were habitual caffeine consumers

Genotype	Native	Heterozygous mutated	Homozygous mutated
CYP1A2*1F rs762551 85 (100 %)	9 (10%) A/A	30 (33%) A/C	51 (57%) C/C
CYP2E1*5A rs3813867 87 (100%)	4 (5%) G/G	21 (23%) G/C	65 (72%) C/C
CYP2E1*5B rs2031920 87 (100%)	67 (75%) C/C	18 (20%) C/T	5 (5%) T/T

Note: *A/A, A/C, C/C* alleles of CYP1A2*1F; *G/G, G/C, C/C* alleles of CYP2E1*5A; *C/C, C/T, TT* alleles of CYP2E1*5B

The prevalence of CYP1A2*1F isoforms was studied in 85 subjects of our population. Overall, among CYP1A2*1F polymorphisms, one tenth of the population was native, a third was heterozygous, and a bit more than half was homozygote mutated. *CYP2E1* polymorphisms were characterized in 87 subjects. Regarding the prevalence of CYP2E1*5A (*rs3813867*) polymorphisms, very few exhibited the native genotype, one fifth genotyped as heterozygous and almost two thirds of the population was homozygote mutated. As to CYP2E1*5B (*rs2031920*) almost three quarters of the population exhibited the native genotype, one sixth was heterozygous, while the minority of the population was found homozygous to the mutated genotype (Table 3).

Comparing our population with the 1000 Genomes Project data, phase III [23], we found that our population is similar to Americans in regards to *CYP1A2 rs762551* polymorphism and significantly different from East Asians, Europeans, Africans, and South Asians (*p* < 0.01). Considering *CYP2E1* polymorphisms *rs3813867*, our population was similar to East Asian and different from African, American, European, and South Asian ancestry populations (*p* < 0.01); as regards to *rs2031920*, our population genotype is similar to American, and different from African, East Asian, European, and South Asian populations. (Table 4).

**Table 4:** Comparative analysis of allelic frequencies of polymorphisms of CYP1A2 and CYP2E1 with other populations

	CYP1A2*1F rs762551			CYP2E1*5A rs3813867			CYP2E1*5B rs2031920		
Population	A	C	*p* †	G	C	*p* †	C	T	*p* †
AFR	0.562	0.438	< 0.0001	0.933	0.067	< 0.0001	0.998	0.002	< 0.0001
AMR	0.758	0.242	0.303	0.878	0.122	0.0073	0.885	0.115	0.0248
EAS	0.673	0.327	0.002	0.798	0.202	0.0375	0.798	0.202	0.0027
EUR	0.680	0.320	0.006	0.959	0.041	< 0.0001	0.959	0.041	< 0.0001
SAS	0.535	0.465	< 0.0001	0.991	0.009	< 0.0001	0.991	0.009	< 0.0001
NEM	0.737	0.263	Ref	0.835	0.165	Ref	0.850	0.150	Ref

(1000 Genomes Project Consortium et al., [23]) †Fisher´s exact test. *AFR* African, *AMR* American, *EAS* East Asian, *EUR* European, *SAS* South Asian, *NEM* Northeast México, *Ref* reference value for studied population. *A and C, G and C, C and T* alleles

Reported allelic frequencies are compared to reference values with Fisher’s exact test

### Correlations of cytochromes metabolic activity and environmental exposures during pregnancy

Cytochrome metabolic activity was expressed as the conversion rate between the product and the substrate. Thus, CYP1A2 activity can be expressed by the paraxanthine-to-caffeine ratio. Accordingly, CYP2E1 activity can be expressed by the theophylline-to-caffeine or theobromine-to-caffeine ratio [5].

When the metabolic activity of CYP1A2 and CYP2E1 was correlated with environmental factors (consumption of charbroiled meat, exposure to carbon fumes, acetaminophen ingestion and alcohol exposure during pregnancy), we found a moderate correlation of consumption of charbroiled meat and increased metabolic activity of CYP2E1, Spaearman’s Rho 0.263(77), *p* = 0.021; the same was true for acetaminophen ingestion, that exhibited a moderate correlation with increased metabolic activity of CYP2E1, Spearman’s Rho 0.256(84), *p* = 0.019.

No correlation was observed between metabolic activity indexes and alcohol exposure during pregnancy. Table 5 shows data on the effect of relevant environmental exposures in pregnant mothers on the metabolic activities of CYP1A2 and CYP2E1.

**Table 5:** Effect of selected environmental factors on cytochromes metabolic activity

	Charbroiled meat			Charcoal fumes			Acetaminophen		
Cytochrome metabolic activity	Exposed (*n* = 46)	Unexposed (*n* = 44)	*p*	Exposed (*n* = 5)	Unexposed (*n* = 85)	*p*	Exposed (*n* = 6)	Unexposed (*n* = 84)	*p*
CYP1A2 ^a^	0.11 (0.02–0.30)	0.05 (0.01–0.16)	NS	0.13 (0.05–0.13)	0.05 (0.02–0.18)	NS	0.05 (0.02–0.09)	0.05 (0.02–0.18)	NS
CYP2E1 ^b^	0.52 (0.18–1.80)	0.26 (0.01–0.81)	† 0.02	0.40 (0.39–1.79)	0.40 (0.02–1.25)	NS	2.0 (1.24–4.46)	0.39 (0.02–1.13)	† 0.02
CYP2E1 ^c^	0.09 (0.02–0.20)	0.05 (0.01–0.15)	NS	0.08 (0.08–0.15)	0.07 (0.02–0.16)	NS	0.12 (0.04–0.20)	0.07 (0.02–0.15)	NS

Values expressed as median (IQR)

†Mann–Whitney’s *U* test; *NS* non-significant *p* value

Metabolic activity:

^a^Paraxanthine/caffeine nanomolar ratio

^b^Theobromine/caffeine nanomolar ratio

^c^Theophylline/Caffeine nanomolar ratio

Significant differences in CYP2E1 metabolic activity were found in mothers who consumed charbroiled meat during pregnancy (half of the study population) compared to those who did not (*p* = 0.02), and as well as in mothers who ingested acetaminophen during pregnancy, despite the small frequency (*p* = 0.02). Consumption of charbroiled meat in the northeast of Mexico is very common, as frequent as twice per week in some families.

**Fig. 3:**
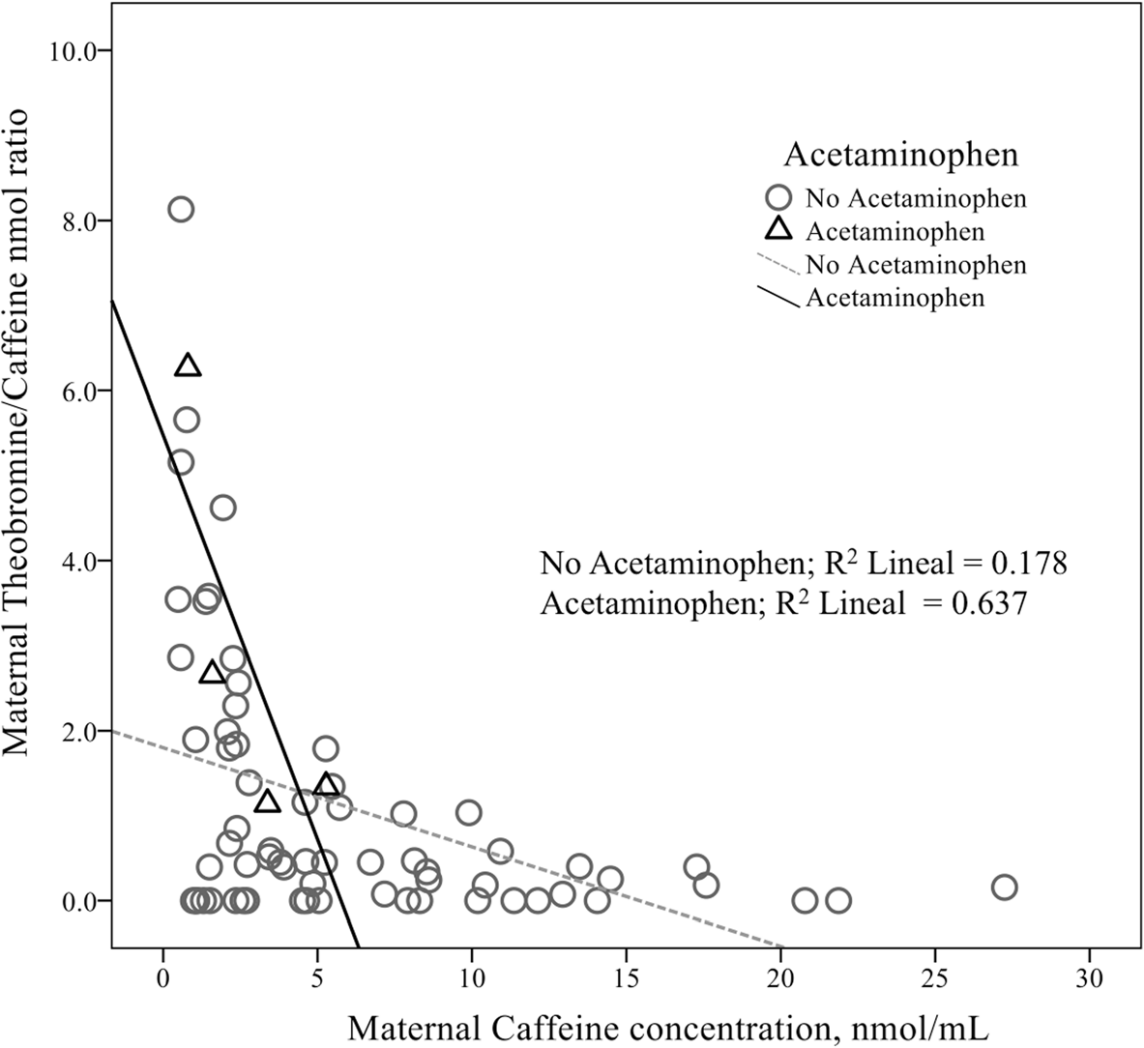
Scatterplot of the maternal theobromine/caffeine nanomolar ratio against maternal caffeine concentration in nanomole/milliliter, surrogate for the metabolic activity rate of CYP2E1. A distinct positive effect on this metabolic activity rate was seen in those subjects exposed to acetaminophen, compared to the rate of those not exposed

Furthermore, in the dispersion plot in Fig. 3, we show the strong effect that the ingestion of acetaminophen exerted in CYP2E1 metabolic activity (theobromine/caffeine ratio), (*R*^2^ = 0.637; *p* < 0.01), compared to that found in those mothers who did not use this medication (*R*^2^ = 0.178).

### Correlation of the rate of cytochromes metabolic activity and pregnancy concurrent events

We explored in search of correlations of gender offspring, gestational diabetes mellitus, pregnancy associated arterial hypertension and administration of prenatal steroids with cytochrome metabolic activity.

Offspring gender exhibited a moderate positive correlation with CYP2E1 associated metabolic activity, and the conversion rate of caffeine to theophylline, Spearman’s Rho 0.236(84), *p* = 0.031. As regards to CYP2E1-associated metabolic activity, we found a moderate positive correlation of pregnancy associated hypertension and the conversion rate of caffeine to theobromine, Spearman’s Rho 0.258(84), *p* = 0.018. The administration of prenatal steroids, confirmed in 68 of the 90 pregnancies, exhibited a moderate negative correlation with the rate of conversion of caffeine to paraxanthine, Spearman’s Rho − 0.234(84), *p* = 0.032. This inhibitory effect was constant for both isoforms of CYP1A2 studied (data not shown).

Table 6 shows data on the effect of pregnancy concurrent events on the metabolic activities of CYP1A2 and CYP2E1.

**Table 6:** Effect of selected pregnancy concurrent events on cytochromes metabolic activity

	Offspring gender (*n* = 98)			Pregnancy associated hypertension			Prenatal steroids		
Cytochrome metabolic activity	Male (*n* = 54)	Female (*n* = 44)	*p*	Exposed (*n* = 10)	Unexposed (*n* = 80)	*p*	Exposed (*n* = 68)	Unexposed (*n* = 22)	*p*
CYP1A2 ^a^	0.05 (0.02–0.16)	0.09 (0.02–0.26)	NS	0.08 (0.02–0.37)	0.05 (0.01–0.18)	NS	0.04 (0.01–0.11)	0.13 (0.03–0.32)	† 0.03
CYP2E1 ^b^	0.35 (0.02–1.24)	0.45 (0.04–1.24)	NS	1.61 (0.66–1.94)	0.37 (0.02–1.12)	† 0.02	0.46 (0.02–1.79)	0.37 (0.05–0.45)	NS
CYP2E1 ^c^	0.04 (0.02–0.11)	0.09 (0.03–0.21)	† 0.03	0.08 (0.02–0.42)	0.07 (0.02–0.15)	NS	0.05 (0.02–0.15)	0.09 (0.02–0.21)	NS

Values expressed as median (IQR)

†Mann–Whitney’s U test; *NS* non-significant *p* value

Metabolic activity:

^a^Paraxanthine/caffeine nanomolar ratio

^b^Theobromine/caffeine nanomolar ratio

^c^Theophylline/caffeine nanomolar ratio

A further partial correlation analysis of the association of pregnancy associated hypertension and those two others previously identified for charbroiled meat and acetaminophen ingestion, with an increased activity of CYP2E1 on the conversion rate of caffeine to theobromine, allowed us to identify that the main correlation was that associated with the ingestion of acetaminophen, partial Rho 0.256(77), *p* = 0.024. A linear regression analysis, with the stepwise method, confirmed the contribution of acetaminophen ingestion to the observed increase in that particular metabolic activity, *F*(1,75) = 5.275, *p* = 0.024, *R*^2^ = 0.07.

## Discussion

Self-reported caffeine consumption (from any known source) was positive in 97% of studied pregnancies. This figure is substantially more than the 57% in the Bracken et al. study [10], the 68% reported in the Frary et al. study [24], and the 64.5% reported for 7607 women and their newborns in the cohort of Ribeirão Preto by Vitti et al. [25]; and with some resemblance to the 84% at 30 weeks of gestation reported in the MoBa cohort by Sengpiel et al. [26] and the 80.1% reported by Santiago et al., in a cohort of predominantly Hispanic pregnant women in California [27]; all these authors relied on self-reporting as the data source. Even after correction, the laboratory confirmed prevalence of caffeine consumption, 93% of the studied population, is higher to all of the above referenced populations. For unknown reasons, our study subjects tended to over-report on caffeine consumption, rendering self-report an inaccurate tool; our data strengthen the concept that a laboratory measurement is the most reliable indicator for caffeine exposure during pregnancy, as Grosso and Bracken have suggested before [4]. Interestingly, some years later, Grosso et al., in 2008, concluded on the contrary, that maternal self reported intake of caffeine was the optimal and most valued measure of antenatal caffeine exposure [28]. Our results offer significant evidence on the value of laboratory measurement of caffeine and metabolites in this particular context.

In our study, the main source of caffeine ingestion was soft-drinks, alone or in combination, as depicted in Fig. 2. In the Frary et al. study [24], the ingestion of soft-drinks was reported by 16% of their subjects, while in the Chen et al. study [29] and the Dott et al. study [30] the percentages varied between 16 and 66 % respectively. In the Błaszczyk-Bebenek et al. study in Southern Poland, the principal source of caffeine was black tea, followed by coffee [31], while in the Lifeways Cross Generation Cohort Study of Chen et al., in Ireland, tea was the predominant source of caffeine (48%), followed by coffee (39%), soft-drinks (8%), and chocolate products (5%) [29].

On a different sociocultural setting, Santiago et al. performed a survey in a cohort of predominantly Hispanic women in California, and found that among caffeine containing beverages consumed during pregnancy, cola soft-drinks were the most popular (60%), followed by coffee (46%) and tea (30%). Nearly half of the women who reported drinking caffeinated beverages did so throughout their pregnancies, while nearly a fifth (19%) only consumed caffeine during the mid- and later parts of their pregnancies [27]. Thus, the frequency and source of ingested caffeine during pregnancy seem to depend strongly on population habits and customs.

In our country, Mexico, the 2016 National Survey on Health and Nutrition [32] has documented that around 85% of the general population report the ingestion of non-dairy sugared beverages (among them coffee and soft-drinks), this figure is identical to the 85% of ingestion of cola soft-drinks alone or in any combination found in our study (Fig. 2).

In the context of current population migration patterns, according to the U.S. Census Bureau, in 2012, Hispanic origin population in the United States of America accounted for at least 17% of the US population [33], so our findings may be of interest not only locally but also in major cities in the US; and perhaps also in other latitudes. The states of California, Texas, Florida, Arizona, New Mexico, New York, New Jersey, and Illinois, contain three-quarters (74%) of the US Hispanic population, that has been dispersing all over the country, since the 1990 census. Nationally, Mexicans in the USA are the largest Hispanic origin group, making up around 65% of all Hispanics immigrants [34].

Pregnancy implies important physiologic and metabolic adaptations. The placenta is responsible for fetal nutrition and, in turn, acts as a barrier to the passage of some substances. A substantial number of expecting mothers drink beverages containing xanthines during pregnancy [2]. Among xanthines, caffeine, a tri-methylxanthine alkaloid, the psychoactive stimulant present in coffee, tea, cola, and energy soft drinks [12] is certainly one of the most common, although it is subject to well described metabolic changes during normal pregnancy [5–8]. In the usual conditions, both caffeine and its metabolites readily cross the placental barrier, and tend to accumulate on the fetal side, since neither the fetus nor the placenta exhibit caffeine metabolic capabilities [9]. The rate of drug transference to the embryo and fetus should be differentiated from the extent of drug transfer because different factors determine these two pharmacokinetic variables [13]. Our results in this topic, F/M ratio, are interesting. We found two distinct patterns of caffeine transplacental transfer; a low transfer rate, and a normal transfer rate. It was interesting that only gestational diabetes mellitus exhibited a clear association with the low transfer rate. The reason behind this fact is not clear for us.

Grosso et al. in 2008 [28] measured caffeine and its main metabolites in maternal urine throughout pregnancy as well as in umbilical cord blood, expressed in μg/mL ± (SD). Although urinary concentrations may not reflect accurately blood concentration, their caffeine values were lower, compared to our findings, measured in blood, 0.78 ± (0.99) vs 1.18 ± (1.15), *p* = 0.014; paraxanthine concentration was higher, 0.83 ± (1.43) vs 0.12 ± (0.17, *p* < 0.0001; theobromine concentration was almost seven times higher, 3.12 ± (4.07) vs 0.46 ± (0.47), *p* < 0.0001; and theophylline concentration was slightly but significantly higher, 0.19 ± (0.26) vs 0.12 ± (0.12), *p* = 0.014. This comparison is subject to due reserve, because the biological source was entirely different.

Concerning cord blood measurements, the same group reported a caffeine concentration very similar to ours, 1.45 ± (1.38) vs 1.28 ± (1.37), *p* = 0.255 (NS). They also found statistically significant higher values for neonatal paraxanthine (0.29 ± 0.25 vs 0.16 ± 0.12, *p* = < 0.001); theobromine levels (0.70 ± 0.79 vs 0.47 ± 0.60 μg/mL, *p* = 0.001); and almost identical theophylline levels (0.12 ± 0.10 vs 0.12 ± 0.12 μg/mL, *p* = 0.7, NS) than those in our study.

Hentges et al. in 2010 [14], in a cohort of 87 preterm neonates, very similar to our neonatal population, reported caffeine concentrations in cord blood; their values, compared to ours were higher (2.3 ± (2.3) vs 1.18 ± (1.11), *p* = 0.001. They did not measure maternal concentration, nor caffeine metabolites.

Wierzejska et al., in 2014 [35], measured caffeine and paraxanthine levels in a cohort of 30 pregnant women and their offspring, born at full term. For maternal caffeine, they report values significantly higher than ours, 3.19 ± (1.6) vs 1.18 (1.15), *p* < 0.0001. For paraxanthine, similar values were observed, 0.72 ± (0.36) vs 0.12 (0.17), *p* < 0.0001. For their at-term neonatal cohort, cord blood caffeine concentrations were significantly lower than ours, 0.36 ± (0.18) vs 1.28 ± 1.37, *p* < 0.0001; while precisely the opposite was true for the paraxanthine values, 0.59 ± (0.29) vs 0.16 ± (0.12), *p* < 0.0001. These last two comparisons suggest that their cohort was actively transforming caffeine to paraxanthine, through the usual CYP1A2 pathway, and may be explained because 20% of their mothers were active smokers during pregnancy. It is known that smoking can enhance CYP1A2 activity [11–13].

None of these authors [14, 28, 35] examined the metabolic activity of microsomal systems, as we did in the present study; perhaps genetic and environmental characteristics in their study population, and ours, may have influenced the results..

We evaluated both CYP1A2 and CYP2E1, the two major caffeine metabolizers in humans, and their variation in the study population. Overall, the frequency of CYP1A2 * 1F alleles (rs762551) was found to be similar to that of other American ancestry, with strong similitude to that of a Chilean mestizo cohort and slightly higher than the Costa Rican and Japanese population [36, 37], but significantly different from the population of Africa, East Asia, European ancestry, and South Asia [22] (see Tables 3 and 4).

In particular, two CYP2E1 polymorphisms were evaluated in our study; rs3813867 (CYP2E1 * 5A), whose frequency was similar to that reported in individuals from East Asia, and significantly different from that reported for African, American, European, and South Asian population described in the Consortium Report of the 1000 Genomes Project, 2015, and rs2031920 (CYP2E1 * 5B), whose frequency was similar to the US population (Hispanic population) and significantly different from that reported from African, East Asian, European, and South population [23] (see Table 4).

CYP1A2 and CYP2E1 are responsible for the first-pass metabolism of endogenous and xenobiotic substances, particularly the first-pass metabolism of caffeine. In pregnancy, starting in the second trimester, metabolic activity of CYP1A2 is reduced by up to 65%, while other metabolic activities such as CYP2E1 are increased [8].

Environmental factors are important because they can enhance or inhibit the activity of a cytochrome. For example, smoking and exposure to volatile aromatic hydrocarbons (tobacco smoke, pollution, and occupational exposure to carbon smoke) induce CYP1A2 activity; while consumption of alcohol and food with nitrosamines (charbroiled meat) and inhalation of volatile aromatic hydrocarbons are important inducers and substrates of CYP2E1. We found that mothers who ingested acetaminophen exhibited higher CYP2E1 metabolic activity (see Table 5) than the unexposed group. In fact, despite that only four of our confirmed caffeine ingesting mothers had exposure to acetaminophen during current pregnancy, this group showed a very clear effect.

CYP2E1 is constitutively expressed in various tissues, such as the liver, skin, placenta and brain [9] and is responsible for the metabolism of xenobiotics, such as the metabolism of alcohol in acetaldehyde, as well as the activation of some procarcinogens (from aromatic hydrocarbons, nitrosamines, etc.) [11]. The metabolism of acetaminophen through this pathway may produce N-acetyl-p-benzoquinone imine [38], which is a toxic compound for neonates. In preterm infants, the glucuronization and sulfidation mechanisms responsible for metabolizing acetaminophen are poorly developed, and the glutathione system, despite being present, has a very limited reaction capacity [39]. This increases the possibility of toxicity to the premature baby if the mother ingests acetaminophen. In Mexico, as well as in the USA and many other countries, acetaminophen is among the pain relievers of choice during pregnancy [40] and is widely available without a prescription. Another possibility arises if acetaminophen is prescribed directly for the neonate, as is increasingly common in current neonatal intensive care practices [41]. That is why we feel that our data will be of interest not only locally, but also in other latitudes, as the USA and Europe, with a strong migration influx of shared ancestry.

Regarding clinical conditions concurrent with pregnancy, such as gestational diabetes mellitus, pregnancy associated hypertension or antenatal steroids usage (see Table 1), we were unable to find any consistent association with the metabolic activity of cytochromes and the resultant caffeine and metabolites concentrations.

At a first glance, the rate of antenatal steroid use in our obstetric population is clearly below international standards, but we must recognize that, although this therapeutic approach has been adopted rather slowly in our country, it has consistently improved in the last decade [42, 43].

The main strenght of this study is the consistency of the eligibility criteria, that allowed us to build a cohort of pregnant mothers, most of them habitual consumers of caffeine containing beverages, who all had a preterm delivery, and kindly accepted the invitation to participate. In this cohort we report results from our studies on CYP1A2 and CYP2E1 metabolic activity, genotyping and the effect of exposure to potential inducers or modifiers of these enzymatic processes in the preterm pregnancy.

As with all observational research designs, the pragmatic nature of the study precludes any conclusion on causality, all positive findings are so only at the association level.

To our knowledge, this is the first study in Mexican population addressing caffeine consumption patterns during preterm pregnancy, and allowed us to establish that caffeine consumption during pregnancy in our population ranks high among worldwide reports on this matter.

Our findings warrant to examine in detail the effects of acetaminophen and other over the counter medications on cytochrome metabolic activity during pregnancy. In the near future, we will explore if this intrauterine exposure exerted any effects on the neonate’s cytochromes metabolic activity.

## Conclusions

The rate of caffeine consumption in our population sample was higher than that reported in other Latin-American populations.

The main sources of caffeine during pregnancy were soft-drinks, alone or in combination with coffee.

Consumption of charbroiled meat and acetaminophen during pregnancy modified the CYP2E1 metabolic activity; this effect seems to be particularly intense with acetaminophen.

The metabolic activation of CYP2E1 interfered with the pharmacokinetics of exogenous caffeine, leading to an increase in the rate of its conversion to theobromine.

## Data Availability

The datasets used and/or analyzed during the current study are available from the corresponding author on reasonable request.
